# The importance of proteinuria and prior cardiovascular disease in all major clinical outcomes of atherosclerotic renovascular disease – a single-center observational study

**DOI:** 10.1186/s12882-016-0409-1

**Published:** 2016-12-07

**Authors:** Diana Vassallo, James Ritchie, Darren Green, Constantina Chrysochou, Joseph Blunt, Philip A. Kalra

**Affiliations:** Department of Renal Medicine, Salford Royal NHS Foundation Trust, Salford, M6 8HD United Kingdom

**Keywords:** Atherosclerotic renovascular disease, Cardiovascular events, End-stage kidney disease, Mortality, Proteinuria, Revascularization

## Abstract

**Background:**

Identification of patients at risk of developing adverse events would enable aggressive medical therapy and possibly targeted revascularization. The aim of this study is to characterize the determinants of long-term outcomes in atherosclerotic renovascular disease (ARVD).

**Methods:**

Patients with a radiological diagnosis of ARVD were recruited into this single-center prospective cohort study between 1986 and 2014. Data collected included baseline co-morbid conditions, annualized prescribed medications and laboratory data (serum creatinine [υmol/L], proteinuria [g/24 h]). Multivariable Cox regression analysis was used to explore association with these end-points: death, end-stage kidney disease (ESKD), cardiovascular event (CVE) and the first of any of these events.

**Results:**

A total of 872 patients were recruited into this study. However, 42 patients were excluded due to missing baseline data and hence case records for 830 patients were reviewed. Over median follow-up of 57.1 months (interquartile range: 21.7–96.9), incidence per 100 patient years of death, ESKD, CVE and any event was 13.5, 4.2, 8.9 and 21.0 respectively. Macrovascular disease (MVD), congestive heart failure (CHF), flash pulmonary oedema (FPE) and greater proteinuria at baseline were individually associated with increased risk for all end-points in multivariable analysis (Death: MVD –HR 1.24 [95% CI 1.02–1.50]; CHF –HR 1.33 [95% CI 1.08–1.64]; FPE – HR 2.10 [95% CI 1.50–2.92]; proteinuria – HR 1.14 [95% CI 1.08–1.20]). Higher estimated glomerular filtration rate at time of diagnosis was significantly associated with reduced risk of all end-points (Death: HR 0.92 [95% CI 0.89–0.94])., Administration of statins and renin angiotensin blockade (RAB) at baseline were also associated with reduced adverse events, especially death (RAB: HR 0.83 [95% CI 0.70–0.98]; statins: HR 0.79 [95% CI 0.66–.94]) and ESKD (RAB: HR 0.84 [95% CI 0.71–1.00]; statins: HR 0.79 [95% CI 0.66–0.93]). Revascularization was associated with reduced risk of death (HR 0.65 [95% CI 0.51–0.83]) and ESKD (HR 0.59 [95% CI 0.46–0.76]).

**Conclusion:**

All patients with ARVD require intensive vascular protection therapy to help mitigate systemic atherosclerosis, optimize cardiovascular risk and improve clinical outcomes. More effort is required to identify the minority of patients who may benefit from revascularization.

## Background

With an increasingly aging population and a rising burden of atherosclerotic risk factors [1], there is a suggestion that atherosclerotic renovascular disease (ARVD) is becoming more prevalent both in the general population and in patients with chronic kidney disease (CKD) [2]. This has important implications because although ARVD is clinically silent in the majority of patients, its presence is independently associated with increased mortality when compared to patients with similar risk factors but no significant renal artery stenosis (hazard ratio 2.9 [risk ratio 1.7–7.0] *p* < 0.0001) [3] or non-ARVD CKD (hazard ratio 1.5 [95% confidence interval 1.2–1.8] *p* < 0.0001) [4]. Only a minority of patients with haemodynamically–significant ARVD present with a ‘high-risk’ clinical phenotype characterized by one or more of uncontrolled hypertension, rapid decline in renal function, and recurrent heart failure [5].

Management of ARVD has been a contentious subject for many years; recent large randomized controlled trials (RCT) have shown that revascularization does not confer added benefit to optimal medical treatment and atherosclerotic risk factor control, the accepted cornerstones of ARVD management [6, 7]. However, as with any RCT, these findings only apply to the type of patients included in the trials; those ARVD patients with high-risk features were seldom recruited into these studies. Anectodal evidence from case reports [8, 9] and more recently, data from an observational single-center study performed by our research group comparing medical treatment with revascularization in 237 patients with a high-risk phenotype, support the role of revascularization in specific clinical situations. In this study we found that revascularization reduced the risk of death in patients presenting with flash pulmonary oedema (hazard ratio 0.4, *p* = 0.01) and was associated with reduced risk of death (hazard ratio 0.15, *p* = 0.04) and cardiovascular events (hazard ratio 0.23, *p* = 0.02) in patients with the combination of refractory hypertension and rapidly declining renal function [10].

Accurate identification of patients with ARVD who are at risk of suffering adverse events would allow a patient-specific therapeutic approach with targeted, intense medical therapy and the possibility of timely revascularization. In this study we utilized clinical and laboratory data acquired over almost 3 decades to characterize the phenotype of patients who reached important clinical end-points, to determine the impact of risk factors on long-term outcomes and assess the effect of revascularization in a large unselected population of patients with ARVD.

## Methods

### Patient population and data collection

All patients with ARVD presenting to our regional renal centre since 1986 have been recruited into this observational epidemiological study. Data were collected on an annual basis from hospital records, in line with ethical approval granted by the local ethics committee and the declaration of Helsinki. Data collection includes baseline demographics (age at diagnosis, gender), co-morbid conditions (diabetes, macrovascular disease [MVD], congestive heart failure [CHF]), presence of flash pulmonary oedema (FPE), and annualized prescribed medications, blood pressure, and laboratory data including serum creatinine (υmol/L) and proteinuria (g/24 h), together with clinical outcome data. The degree of renal artery stenosis (RAS) was obtained from cross-sectional angiography (intravenous digital subtraction angiography [IVDSA] and intra-arterial digital subtraction angiography [IADSA] in earlier studies, computed tomographic [CT] or magnetic resonance [MR] angiography in later studies), reported largely by two specialist radiologists over a thirty year period, and recorded using a ‘patency score’; a score of 200 was equivalent to 0% bilateral stenosis while a score of 0 meant 100% bilateral occlusion. The date of diagnostic imaging was considered as time zero for study entry. Sequential patients were entered into the database up until 31^st^ August 2014 and data censoring was performed at the earliest of 11^th^ May 2015, death, or last patient encounter if discharged or lost to follow-up.

### Definitions

Previous MVD was defined as a composite of documented coronary artery disease (symptomatic angina, previous myocardial infarction or coronary artery bypass grafting, positive coronary angiography or exercise stress test result), peripheral vascular disease (symptomatic intermittent claudication, previous peripheral revascularization, amputation due to limb ischaemia, evidence of ischaemic ulcers or gangrene) and aortic abdominal aneurysms (AAA) confirmed on imaging or previous AAA repair. CHF was defined as documented symptoms of orthopneoa, paroxysmal nocturnal dyspnea, clinical evidence of CHF on examination and/or echocardiographic left ventricular ejection fraction <40%. FPE was defined as acute decompensated heart failure in the absence of a documented precipitating cardiac event or known reduced ejection fraction (<40%). Estimated glomerular filtration rate (eGFR) was calculated using the Chronic Kidney Disease Epidemiology Collaboration equation (CKD-EPI) [11].

### Patient management

Patients were managed in accordance with the contemporary vascular protective advice and UK Renal Association blood pressure targets [12, 13]. Renal revascularization was performed in accordance with physician preference or after entry into a randomized trial [6, 7]. All revascularization procedures involved percutaneous transluminal angioplasty with or without deployment of bare-metal stents; no embolic protection devices were used.

### Clinical end-points

Predefined primary clinical end-points include:

1. Date of death as documented on hospital records. This included all causes of death.
2. Date of first cardiovascular event (CVE) after enrollment, a composite of acute coronary syndrome or myocardial infarction, new arrhythmias, pulmonary oedema or decompensated heart failure, cerebrovascular events including transient ischaemic attacks, peripheral vascular disease including peripheral revascularization and abdominal aortic aneurysm repair, and mesenteric ischaemia.
3. Date of reaching ESKD defined as the earliest of the following events: initiation of renal replacement therapy (RRT) (including renal transplantation) or reaching eGFR <10 ml/min/1.73 m^2^ which is the average eGFR at which RRT is started in the UK [14].
4. A composite end-point composed of the first of any of the above events.

### Statistical analysis

Demographic features, imaging characteristics of ARVD, comorbid conditions, baseline medications, blood pressure, eGFR, proteinuria and rate of eGFR change were compared between patients who reached clinical end-points (death, ESKD, CVE or any event) and those who did not suffer these adverse events. Non-parametric continuous variables are presented as median (interquartile range). Chi-squared test was used to compare categorical data between the two groups while Mann–Whitney-U was used for non-parametric continuous data. The rate of change of eGFR or eGFR slope from time zero to end of study was calculated from slope of linear regression, using serial serum creatinine measurements. Patients who had blood results taken during in-patient stay, patients who reached RRT, and patients with less than 1 year follow-up or less than 3 serum creatinine measurements were excluded from the analysis. For revascularized patients, the rate of change of eGFR or eGFR slope was calculated from at least three pre-revascularization serum creatinine values only. Unadjusted incidence rates per 100 patient years were calculated manually using the following equation: (total number of events/total follow-up time) × 100. Baseline variables were entered into a univariable and multivariable Cox Proportional Hazards model to determine hazard ratios and 95% confidence intervals; variables were entered into the multivariable model depending on clinical plausibility of causal association with outcome and non-adjusted statistical significance. A *P*-value <0.05 was considered to be statistically significant. Continuous variables were centered around the mean and scaled where clinically appropriate. These analyses were performed using SPSS (version 22.0).

## Results

A total of 872 patients were recruited into this observational study; 42 (4.8%) patients were excluded due to one or more missing key baseline parameters (medications [*n* = 4], blood pressure [*n* = 15], eGFR [*n* = 5] and proteinuria [*n* = 25]), leaving a study population of 830 patients with complete datasets.

Median age was 71.0 years (interquartile range: 64.8–76.7). Unilateral ≥70% RAS with contralateral <70% RAS was present in 338 patients (39.5%) while 88 patients (10.6%) had ≥70% RAS bilaterally. At time of ARVD diagnosis, 71.8% of patients had evidence of extra-renal atherosclerosis, 50.0% were receiving an angiotensin converting enzyme inhibitor (ACEi) or angiotensin receptor blocker (ARB) 37.1% a beta blocker, 54.5% aspirin and 55.9% a statin (Table 1).

**Table 1:** Baseline characteristics for entire cohort and for patients who reached and did not reach end-points

	All	Died			ESKD			CVE			Any			Test
	*n* = 830	No (*n* = 226)	Yes (*n* = 604)	*p*	No (*n* = 658)	Yes (*n* = 172)	*p*	No (*n* = 520)	Yes (*n* = 310)	*p*	No (*n* = 148)	Yes (*n* = 682)	*p*	
Median age (years)	71.0 (64.8–76.7)	**68.6**	**72.1**	<**0.0001**	71.5	70.2	0.1	71.4	70.4	0.1	**69.1**	**71.4**	**0.01**	MWU
Male (%)	59.9	59.3	60.1	0.8	59	63.4	0.3	58.1	62.9	0.2	57.4	60.4	0.5	*X* ^2^
RAS >70% unilateral (%)	39.5	35.0	41.2	0.1	38.8	42.4	0.4	41.5	36.1	0.1	35.1	40.5	0.2	*X* ^2^
RAS >70% Bilateral (%)	10.6	9.3	11.1	0.5	10.3	11.6	0.6	10.6	10.6	0.9	6.8	11.4	0.09	*X* ^2^
Median patency score	105.0 (70.0–150.0)	**120.0**	**100.0**	**0.007**	110.0	100.0	0.1	105.0	107.5	0.9	**130.0**	**100.0**	<**0.0001**	MWU
Median SBP mmHg	152.0 (135.0–175.3)	152.0	152.0	0.8	154.0	150.0	0.2	150.0	153.5	0.6	150.0	152.5	0.6	MWU
Median DBP mmHg	80.0 (70.0–90.0)	79.0	80.0	0.3	80.0	80.0	0.9	80.0	80.0	0.9	79.0	80.0	0.2	MWU
Median MAP mmHg	103.3 (93.3–115.3)	101.8	103.3	0.5	103.3	101.4	0.5	102.8	103.3	0.8	100.7	103.3	0.3	MWU
MVD (%)	71.8	**63.3**	**75.0**	**0.001**	71.7	72.1	0.9	68.5	**77.4**	**0.006**	**60.1**	**74.3**	<**0.0001**	*X* ^2^
CHF (%)	19.4	**11.1**	**22.5**	<**0.0001**	19.0	20.9	0.6	**17.3**	22.9	0.05	**8.1**	**21.8**	<**0.0001**	*X* ^2^
FPE (%)	6.4	4.4	7.1	0.2	6.2	7.0	0.7	5.6	7.7	0.2	4.1	6.9	0.2	*X* ^2^
Diabetes (%)	31.3	28.3	32.5	0.2	30.5	34.3	0.3	30.2	33.2	0.4	29.1	31.8	0.5	*X* ^2^
RAB (%)	50.0	**66.4**	**43.9**	<**0.0001**	**52.1**	**41.9**	**0.02**	49.6	50.6	0.8	**64.9**	**46.8**	<**0.0001**	*X* ^2^
BB (%)	37.1	**43.4**	**34.8**	**0.02**	35.9	41.9	0.1	37.5	36.5	0.8	**44.6**	**35.5**	**0.04**	*X* ^2^
CaB (%)	55.4	52.7	56.5	0.3	**52.7**	**65.7**	**0.002**	54.4	57.1	0.5	48.6	56.9	0.07	*X* ^2^
>3 anti-hypertensives (%)	48.1	**54.9**	**45.5**	**0.02**	47.6	50.0	0.6	45.8	51.9	0.09	53.4	46.9	0.2	*X* ^2^
Aspirin (%)	54.5	54.9	54.3	0.9	54.0	56.4	0.6	50.6	**61.0**	**0.004**	52.0	55.0	0.5	*X* ^2^
Statin (%)	55.9	**69.9**	**50.7**	<**0.0001**	56.1	55.2	0.8	**54.8**	57.7	0.4	**68.2**	**53.2**	**0.001**	*X* ^2^
Median Proteinuria (g/day)	0.6 (0.2–1.2)	**0.3**	**0.6**	<**0.0001**	**0.4**	**1.0**	<**0.0001**	0.5	0.6	0.2	**0.2**	**0.6**	<**0.0001**	MWU
Median eGFR^a^ (ml/min/1.73 m^2^)	29.9 (19.3–43.2)	**36.8**	**27.3**	<**0.0001**	**33.4**	**17.4**	<**0.0001**	29.5	30.8	0.3	**37.6**	**28.0**	<**0.0001**	MWU
Revascularized (%)	17.5	19.9	16.6	0.3	16.9	19.8	0.4	**14.8**	**21.9**	**0.009**	13.5	18.3	0.2	*X* ^2^

*BB* beta blocker, *CaB* calcium channel blocker, *CHF* congestive heart failure, *CVE* cardiovascular event, *DBP* diastolic blood pressure, *eGFR* estimated glomerular filtration rate, *ESKD* end-stage kidney disease, *FPE* flash pulmonary oedema, *MAP* mean arterial pressure, *MVD* macrovascular disease, *MWU* Mann Whitney *U* test, *n* number of patients, *RAB* renin-angiotensin blockade, *RAS* renal artery stenosis, *SBP* systolic blood pressure, *X* ^2^ chi-square test. Bold data indicates a statistically significant difference with a p value less than 0.05

^a^Calculated using Chronic Kidney Disease Epidemiology collaboration equation (CKD-EPI)^11^

Over a median follow-up of 57.1 months (interquartile range: 21.7–96.9 months), 604 (72.8%) patients died, 172 (20.7%) reached ESKD (of whom 128 [15.4%] were treated with RRT), 310 (37.3%) suffered a CVE (of whom 46 [14.8%] suffered a fatal CVE) and 682 (82.2%) experienced any of the previous events. In total, 145 (17.5%) patients underwent renal revascularization. The incidence per 100 patient years of death, ESKD, CVE and any event was 13.5, 4.3, 8.9 and 21.0 respectively.

Patients who died were older than surviving patients (72.1 versus 68.6 years, *p* < 0.0001), had higher prevalence of MVD (75.0% versus 63.3%, *p* = 0.001) and CHF (22.5% versus 11.1%, *p* < 0.0001) at baseline, and were less likely to be receiving renin-angiotensin blockade (RAB) (43.9% versus 66.4%, *p* < 0.0001), more than 3 anti-hypertensive agents (45.5% versus 54.9%, *p* = 0.017) or statins (50.7% versus 69.9%, *p* < 0.0001) at time of diagnosis. These patients were also noted to have lower patency score (100.0 versus 120.0, *p* = 0.007), greater degree of proteinuria (0.6 versus 0.3 g/day, *p* < 0.0001) and a lower eGFR (27.3 versus 36.8 ml/min/1.73 m^2^, *p* < 0.0001) at baseline. Patients who reached ESKD similarly had more proteinuria (1.0 versus 0.4 g/day, *p* < 0.0001) and worse CKD (eGFR 17.4 versus 33.4 ml/min/1.73 m^2^, *p* < 0.0001) at time of diagnosis. Comorbidities and baseline medications were similar between patients who reached ESKD and those who did not, although a higher proportion of patients who suffered ESKD were receiving calcium channel blockers at time of diagnosis (65.7% versus 52.7%, *p* = 0.002) and conversely, less patients who reached ESKD were established on an ACEi or an ARB (41.9% versus 52.1%, *p* = 0.02) (Table 1).

Patients who suffered a CVE were more likely to have had revascularization compared to patients who remained CVE-free (21.9% versus 14.8%, *p* = 0.009), and they were more likely to be established on Aspirin at time of diagnosis (61.0% versus 50.6%, *p* = 0.004). Patients who reached the composite end-point of any of ESKD, CVE events or mortality were enriched with extra-renal atherosclerosis and cardiovascular disease at baseline, and a fewer proportion were established on vasculoprotective therapy. Degree of stenosis, CKD and proteinuria were significantly worse when compared to those who did not achieve these end-points. Rate of loss of eGFR was also significantly faster in patients who reached ESKD (−2.0 versus−0.6 ml/min/1.73 m^2^/year, p <0.0001), suffered a CVE (−1.2 versus−0.7 ml/min/1.73 m^2^/year, *p* = 0.02) or any event (−1.0 versus−0.4 ml/min/1.73 m^2^/year, *p* = 0.01) (Table 2).

**Table 2:** Rate of eGFR decline per year for patients who reached clinical end-points and those who remained event-free

	All			Died			ESKD			CVE			Any		
				Yes (*n* = 400)	No (*n* = 201)	*p*	Yes (*n* = 108)	No (*n* = 493)	*p*	Yes (*n* = 244)	No (*n* = 357)	p	Yes (*n* = 469)	No (*n* = 132)	*p*
Median eGFR slope^a^ (ml/min/1.73 m^2^/year)	(*n* = 601) −0.9 (−3.0–0.9)			−1.1 (−3.5–1.2)	−0.6 (−2.2–0.8)	0.09	−2.0 (−4.6–−0.8)	−0.6 (−2.5–1.4)	<0.0001	−1.2 (−3.3–0.7)	−0.7 (−2.6–1.5)	0.02	−1.0 (−3.4–0.9)	−0.4 (−1.8–0.9)	0.01
					Died (*n* = 400)			ESKD (*n* = 108)			CVE (*n* = 244)			Any (*n* = 469)	
**Revascularization status**	**NR** (***n*** = **517**)	**R** (***n*** = **84**)	***p***	**NR** (***n*** = **347**)	**R** (***n*** = **53**)	***p***	**NR** (***n*** = **91**)	**R** (***n*** = **17**)	***p***	**NR** (***n*** = **201**)	**R** (***n*** = **43**)	***p***	**NR** (***n*** = **397**)	**R** (***n*** = **72**)	***p***
**Median eGFR slope** ^a^(**ml**/**min**/**1.73 m** ^**2**^/**year**)	−0.8 (−2.6–0.9)	−1.7 (−9.8–−1.7)	0.3	−1.0 (−3.2–0.9)	−1.5 (−11.2–7.6)	0.9	−2.1 (−4.6–−0.9)	−1.9 (−5.8–3.1)	0.9	−1.2 (−3.1–0.3)	1.4 (−9.3–5.9)	0.6	−1.0 (−3.2–0.9)	−1.6 (−9.8–5.8)	0.8

*CVE* cardiovascular event, *eGFR* estimated glomerular filtration rate, *eGFR* estimated glomerular filtration rate calculated using Chronic Kidney Disease Epidemiology collaboration equation (CKD-EPI)^11^, *ESKD* end-stage kidney disease, *MWU* Mann Whitney *U* Test, *n* number of patients who met criteria for calculation of eGFR slope, *NR* non-revascularized, *R* revascularized. Bold data indicates a statistically significant difference with a p value less than 0.05

^a^Representing rate of eGFR decline per year. This was calculated from slope of linear regression, excluding blood results taken during in-patient stay, patients who reached RRT, and patients with less than 1 year follow-up or less than 3 data points. For revascularized patients, only pre-revascularization serum creatinine values were entered into the analysis

Table 3 compares baseline characteristics between patients who underwent revascularization and those who were treated exclusively medically; as expected, overall, revascularized patients had more severe stenosis with more frequent bilateral severe disease. A higher proportion of these patients also had documented cardiovascular disease and evidence of heart failure at time of diagnosis. Baseline renal function, degree of proteinuria and rate of eGFR decline (Table 2) did not differ between revascularized and non-revascularized patients.

**Table 3:** Comparison of baseline characteristics between revascularized and non-revascularized patients

	All (*n* = 830)			Death (*n* = 604)			ESKD (*n* = 172)			CVE (*n* = 310)			Any (*n* = 682)		
	Non-revascularized (*n* = 685)	Revascularized (*n* = 145)	*p*	Non-revascularized (*n* = 504)	Revascularized (*n* = 100)	*p*	Non-revascularized (*n* = 138)	Revascularized (*n* = 34)	*p*	Non-revascularized (*n* = 242)	Revascularized (*n* = 68)	*p*	Non-revascularized (*n* = 557)	Revascularized (*n* = 125)	*p*
Median age (years)	**71.6** (**65.1**–**77.3**)	**69.2** (**63.3**–**74.6**)	**0.001**	**72.1** (**67.1**–**78.1**)	**69.5** (**65.2**–**75.1**)	**0.002**	70.4 (64.1–75.3)	68.5 (62.6–75.8)	0.4	**71.3** (**65.2**–**76.7**)	**68.5** (**62.8**–**73.1**)	**0.007**	**71.9** (**66.4**–**77.4**)	**69.2** (**64.0**–**64.5**)	<**0.0001**
Male (%)	61.2	53.8	0.1	61.5	53.0	0.1	64.5	58.8	0.5	64.0	58.8	0.4	61.2	56.8	0.4
RAS >70% unilateral (%)	38.2	45.5	0.1	40.5	45.0	0.4	42.8	41.2	0.9	35.1	39.7	0.5	39.7	44.0	0.4
RAS >70% Bilateral (%)	**6.6**	**29.7**	**<0.0001**	**7.3**	**30.0**	<**0.0001**	**7.2**	**29.4**	<**0.0001**	**5.8**	**27.9**	<**0.0001**	**7.5**	**28.8**	<**0.0001**
Median patency score	**110.0** (**90.0**–**150.0**)	**75.0** (**40.0**–**120.0**)	<**0.0001**	**100.0** (**80.0**–**150.0**)	**72.5** (**40.0**–**115.0**)	<**0.0001**	**100.0** (**73.8**–**150.0**)	**82.5** (**40.0**–**120.0**)	**0.005**	**120.0** (**80.0**–**150.0**)	**75.0** (**40.0**–**110.0**)	<**0.0001**	**105.0** (**132.0**–**172.5**)	**75.0** (**40.0**–**120.0**)	<**0.0001**
Median SBP (mmHg)	150.0 (133.5–172.0)	160.0 (139.0–186.0)	**0.003**	**150.0** (**132.3**–**174.5**)	**160.0** (**140.0**–**185.8**)	**0.03**	146.5 (132.0–166.8)	156.5 (140.0–177.8)	0.08	151.7 (134.8–175.0)	160.0 (139.3–185.8)	0.09	**150.0** (**132.0**–**172.5**)	**160.0** (**140.0**–**186.0**)	**0.004**
Median DBP (mmHg)	**80.0** (**70.0**–**90.0**)	**80.0** (**72.0**–**90.0**)	0.06	80.0 (70.0–90.0)	80.0 (72.0–89.5)	0.2	80.0 (70.0–86.0)	80.0 (70.0–86.5)	0.4	80.0 (70.0–89.0)	80.0 (75.0–89.5)	0.1	80.0 (70.0–90.0)	80.0 (72.7–90.0)	0.08
Median MAP (mmHg)	102.3 (93.0–114.3)	106.7 (96.7–120.0)	**0.007**	103.3 (92.7–116.6)	106.7 (96.7–118.1)	0.05	100.2 (93.2–113.1)	106.2 (96.6–118.0)	0.2	103.3 (93.3–114.2)	105.0 (96.8–120.5)	0.08	**103.3** (**92.5**–**115.6**)	**106.7** (**96.7**–**120.0**)	**0.01**
MVD (%)	70.4	78.6	0.05	73.6	82.0	0.08	71.7	73.5	0.8	75.2	85.3	0.08	**72.7**	**81.6**	**0.04**
CHF (%)	**17.4**	**29.0**	**0.001**	**20.6**	**32.0**	**0.01**	19.6	26.5	0.4	21.5	27.9	0.3	**19.9**	**30.4**	**0.01**
FPE (%)	**5.4**	**11.0**	**0.01**	6.3	11.0	0.1	6.5	8.8	0.6	7.0	10.3	0.4	**6.1**	10.4	0.09
Diabetes (%)	31.4	31.0	**0.9**	32.3	33.0	0.9	34.1	35.3	0.9	36.0	23.5	0.06	32.3	29.6	0.6
RAB (%)	49.9	50.3	0.9	43.5	46.0	0.6	41.3	44.1	0.8	51.7	47.1	0.5	46.5	48.0	0.8
BB (%)	36.2	41.4	0.2	34.3	37.0	0.6	40.6	47.1	0.5	35.5	39.7	0.5	34.5	40.0	0.2
CaB (%)	54.7	58.6	0.4	55.2	63.0	0.1	65.2	67.6	0.8	56.6	58.8	0.7	55.7	62.4	0.2
>3 anti-hypertensives (%)	47.3	51.7	0.3	45.2	47.0	0.7	50.7	47.1	0.7	53.3	47.1	0.4	46.5	48.8	0.6
Aspirin (%)	53.3	60.0	0.1	52.8	62.0	0.09	54.3	64.7	0.3	58.3	70.6	0.07	53.3	62.4	0.07
Statin (%)	55.3	58.6	0.5	49.8	55.0	0.3	53.6	61.8	0.4	58.3	55.9	0.7	52.4	56.8	0.4
Median Proteinuria (g/day)	0.6 (0.2–1.2)	0.4 (0.2–1.1)	0.3	0.6 (0.2–1.3)	0.5 (0.2–1.2)	0.2	1.0 (0.6–1.9)	0.7 (0.3–1.4)	0.1	0.6 (0.2–1.1)	0.6 (0.2–1.1)	0.4	0.6 (0.2–1.3)	0.5 (0.2–1.1)	0.1
Median eGFR^a^ (ml/min/1.73 m^2^)	29.9 (18.8–43.2)	30.2 (20.0–43.4)	0.7	27.2 (16.7–40.5)	28.5 (17.3–37.7)	0.9	16.0 (9.3–26.8)	21.5 (10.3–30.5)	0.2	29.9 (19.4–44.5)	34.1 (23.0–45.4)	0.2	27.4 (17.2–41.3)	29.5 (19.6–40.1)	0.3

*BB* beta blocker, *CaB* calcium channel blocker, *CHF* congestive heart failure, *CVE* cardiovascular event, *DBP* diastolic blood pressure, *eGFR* estimated glomerular filtration rate, *ESKD* end-stage kidney disease, *FPE* flash pulmonary oedema, *MAP* mean arterial pressure, *MVD* macrovascular disease, *MWU* Mann Whitney *U* test, *n* number of patients, *RAB* renin-angiotensin blockade, *RAS* renal artery stenosis, *SBP* systolic blood pressure, *X* ^2^ Chi-square test. Bold data indicates a statistically significant difference with a p value less than 0.05

^a^Calculated using Chronic Kidney Disease Epidemiology collaboration equation (CKD-EPI)^11^

Both univariable and multivariable analysis revealed that MVD, CHF, FPE and higher proteinuria at baseline increased the risk for all four end-points (Table 4). Conversely, better eGFR at time of diagnosis, higher patency score, use of vascular protection therapy and revascularization reduced risk of adverse events. In the adjusted multivariable analysis, administration of statins and renin angiotensin blockade (RAB) at time of diagnosis was associated with reduced risk of death (RAB: hazard ration 0.83 [95% CI 0.70–0.98]; statins: hazard ratio 0.79 [95% CI 0.66–0.94]) and ESKD (RAB: hazard ratio 0.84 [95% CI 0.71–1.00]; statins: hazard ratio 0.79 [95% confidence interval 0.66–0.93]). Renin angiotensin blockade was associated with reduced risk of CVE and any event in univariable analysis but lost significance in multivariable analysis. Similarly, beta-blocker administration at baseline was associated with reduced hazard ratios for adverse events in the univariable analysis, but did not reach statistical significance in the adjusted analysis. Revascularization appeared to significantly reduce the risk for both death (hazard ratio 0.65 [95% confidence interval 0.51–0.83]) and ESKD (hazard ratio 0.59 [95% confidence interval 0.46–0.76]) (Table 4).

**Table 4:** Univariable and multivariable association between baseline variable and clinical end-points

	Death				ESKD^a^				CVE^a^				Any			
	Univariable		Multivariable		Univariable		Multivariable		Univariable		Multivariable		Univariable		Multivariable	
	HR	95% CI	HR	95% CI	HR	95% CI	HR	95% CI	HR	95% CI	HR	95% CI	HR	95% CI	HR	95% CI
Age^b^	1.48	(1.34–1.63)	**1.38**	(**1.24**–**1.54**)	1.36	(1.23–1.50)	**1.17**	(**1.06**–**1.31**)	1.29	(1.18–1.41)	**1.21**	(**1.10**–**1.33**)	1.22	1.12–1.33	1.09	(0.99–1.21)
Patency score^c^	0.95	(0.90–0.99)	0.95	(0.90–1.00)	0.95	(0.90–0.98)	**0.95**	(**0.90**–**0.98**)	0.93	(0.90–0.98)	0.95	(0.93–1.01)	0.93	0.90–0.98	0.95	(0.93–1.00)
Revascularization	0.72	(0.58–0.90)	**0.65**	(**0.51**–**0.83**)	0.70	(0.57–0.87)	**0.59**	(**0.46**–**0.76**)	0.89	(0.73–1.09)	−	−	0.92	0.76–1.12	−	−
MVD	1.44	(1.20–1.73)	**1.24**	(**1.02**–**1.50**)	1.37	(1.15–1.65)	1.17	(0.97–1.42)	1.61	(1.35–1.91)	**1.38**	(**1.15**–**1.65**)	1.50	1.26–1.79	**1.27**	(**1.06**–**1.52**)
Diabetes	1.17	(0.99–1.39)	1.07	(0.89–1.28)	1.09	(0.92–1.30)	0.98	(0.81–1.17)	1.20	(1.02–1.41)	1.02	(0.86–1.21)	1.13	0.96–1.33	0.94	(0.79–1.12)
CHF	1.74	(1.43–2.11)	**1.33**	(**1.08**–**1.64**)	1.77	(1.46–2.14)	1.39	(1.13–1.71)	1.84	(1.53–2.21)	**1.37**	(**1.11**–**1.68**)	1.89	1.57–2.27	**1.42**	(**1.16**–**1.75**)
FPE	2.13	(1.56–2.91)	**2.10**	(**1.50**–**2.92**)	2.01	(1.48–2.73)	**1.82**	(**1.31**–**2.51**)	2.12	(1.56–2.88)	**1.88**	(**1.36**–**2.60**)	2.05	1.51–2.77	**1.72**	(**1.24**–**2.38**)
RAB	0.76	(0.65–0.89)	**0.83**	(**0.70**–**0.98**)	0.78	(0.66–0.92)	**0.84**	(**0.71**–**1.00**)	0.85	(0.73–0.99)	0.88	(0.75–1.03)	0.83	0.71–0.96	0.85	(0.73–1.00)
BB	0.84	(0.71–0.99)	0.93	(0.78–1.10)	0.85	(0.72–1.00)	0.89	(0.75–1.05)	0.84	(0.72–0.99)	0.90	(0.76–1.06)	0.88	0.75–1.03	0.92	(0.78–1.08)
Statin	0.80	(0.68–0.95)	**0.79**	(**0.66**–**0.94**)	0.81	(0.69–0.95)	**0.79**	(**0.66**–**0.93**)	0.93	(0.80–1.08)	0.92	(0.78–1.08)	0.91	0.78–1.06	0.90	(0.76–1.06)
MAP^d^	0.94	(0.90–0.98)	0.97	(0.92–1.02)	0.93	(0.90–0.98)	0.95	(0.90–1.00)	0.93	(0.90–0.98)	0.95	(0.91–1.00)	0.94	0.90–0.98	**0.94**	(**0.90**–**0.99**)
Proteinuria^e^ (g/day)	1.12	(1.07–1.18)	**1.14**	(**1.08**–**1.20**)	1.13	(1.08–1.18)	**1.14**	(**1.09**–**1.20**)	1.09	(1.04–1.14)	**1.10**	(**1.05**–**1.16**)	1.11	1.06–1.16	**1.12**	(**1.07**–**1.18**)
eGFR^f^ (ml/min/1.73 m^2^)	0.89	(0.87–0.91)	**0.92**	(**0.89**–**0.94**)	0.86	(0.84–0.89)	**0.89**	(**0.86**–**0.91**)	0.92	(0.89–0.94)	**0.94**	(**0.92**–**0.97**)	0.90	0.88–0.92	**0.92**	(**0.89**–**0.94**)

*BB* beta blocker, *CHF* congestive heart failure, *CI* confidence interval, *CVE* cardiovascular event, *eGFR* estimated glomerular filtration rate, *ESKD* end-stage kidney disease, *FPE* flash pulmonary oedema, *HR* hazard ratio, *MAP* mean arterial pressure, *MVD* macrovascular disease, *RAB* renin-angiotensin blockade. Bold data indicates a statistically significant association with a p value less than 0.05

^a^Adjusted for death

^b^Per 10 year increase

^c^Per 25 unit increase in patency score

^d^Per 10 mmHg increase in MAP

^e^Per 1 g/day increase in proteinuria

^f^Per 5 ml/min/1.73 m^2^ increase in eGFR, calculated using the Chronic Kidney Disease Epidemiology collaboration equation (CKD-EPI)^11^

## Discussion

This observational study is characterized by the longest follow-up on the largest cohort of patients with ARVD to date, thus providing important insight into the determinants of long-term outcomes.

ARVD occurs as part of systemic atherosclerosis hence, as expected, the phenotype of patients who reached clinical end-points is enriched with typical cardiovascular risk factors such as older age, co-existing macrovascular disease and congestive heart failure [3, 15, 16]. In spite of this, in our center only around half of patients were established on vascular protective therapy at time of diagnosis; this proportion appears to be less than recent data published by the Cardiovascular outcomes in renal atherosclerotic lesions (CORAL) study group, showing that in recruiting centers outside the US, up to 75% of patients were established on statins, and 62% were receiving RAB at baseline [17]. However, our study includes data from a minority of patients recruited before the emergence of evidence on the benefits of vascular protection and tight cardiovascular risk factor control in patients with systemic atherosclerosis [18, 19]. Data from retrospective observational studies has consistently shown that RAB, statins, and more recently, anti-platelet therapy and beta-blockers, offer a similar prognostic benefit to patients with ARVD [20–25]. Although adoption of this multi-targeted therapeutic approach in treating patients with ARVD has increased in recent years, more effort is required to ensure that this becomes standard care for all patients with ARVD.

While our results suggest that patients who reached adverse end-points were less likely to be receiving vascular protective medication, in multivariable analysis only baseline administration of statins was shown to exert an independent mortality benefit. Contrary to expectations, statins had no impact on risk of CVE either in univariable or multivariable analysis and neither RAB nor beta-blockers were significantly associated with benefit in the adjusted analysis. This lack of perceived benefit is probably due to both immortal time bias, as the duration of time patients were receiving the baseline drugs before recruitment into the study was not considered, and the absence of longitudinal drug data. Selection bias may also account for the larger proportion of patients who suffered a CVE that were administered aspirin at baseline due to their higher cardiovascular risk, and the frequent use of calcium channel blockers in patients progressing to ESKD. Indeed, patients who died or reached ESKD had significantly lower renal function at time of diagnosis, hence they were less likely to receive renin-angiotensin blockade for blood pressure control or amelioration of proteinuria.

Although this study suggests that vascular protective therapy may modulate adverse outcomes in ARVD, our results clearly show that these are strongly dictated by the presence of greater degrees of proteinuria and lower GFR, markers of prior renal intrarenal injury. This is in keeping with results published previously by our study group showing that relative risk of declining renal function was 1.23 for every 1 g/24 h increase in baseline proteinuria [26], and patients with >0.6 g/24 h proteinuria at time of diagnosis experienced poor renal outcomes even after revascularization [27]. Low eGFR at baseline was also associated with poor survival [26, 28]. Our study provides further support to this data by showing that patients with greater degrees of baseline proteinuria were at greater risk of suffering all adverse events, while patients with better preserved renal function at time of diagnosis had better outcomes. Although diabetics have been included in the analysis and diabetic nephropathy is also associated with proteinuria, the presence of diabetes did not enhance the risk of adverse clinical outcomes. A recent observational study from Taiwan has reported that diabetes increased the risk of ESKD in ARVD around 1.55-fold [29] while another study from the UK has highlighted increased mortality in diabetic patients with ARVD compared to their non-diabetic counterparts [30]. Potential reasons for our conflicting results are the uniform distribution of diabetics between groups, and selection bias; patients with rapidly declining renal function or severe proteinuria in the context of presumed significant diabetic nephropathy are unlikely to be referred for investigation of ARVD, despite the known close association between diabetes and the development of systemic and renal atherosclerosis [31, 32].

The clinical significance of renal vascular anatomy is unclear. Several studies, including publications from this same dataset, demonstrated that severity of stenosis correlates inversely with long-term patient survival [15, 26, 30], but has no bearing on degree of renal dysfunction at presentation and renal functional outcome. This is dependent on the degree of parenchymal disease, which is related to the actual ‘haemodynamic significance’ of a stenosis rather than its ‘severity’ on cross-sectional imaging studies [26, 28, 33]. In addition, patients with severe stenosis invariably have widespread systemic atherosclerosis and significant cardiovascular comborbidities, hence most die before progressing to ESKD. Our results point towards a trend between higher patency score and better long-term clinical outcomes, suggesting that in this complex, heterogenous condition, outcomes are influenced by both parenchymal damage and the ‘haemodynamic significance’ of stenosis. However, the patency score used in this analysis does not distinguish between unilateral severe stenosis and bilateral less haemodynamically significant disease, hence results probably reflect the effect of overall atherosclerotic burden rather than specific haemodynamic compromise.

Nonetheless, revascularization was noted to exert a significant beneficial effect on long-term survival and progression to ESKD even after adjusting for confounders including age, macrovascular disease, congestive heart failure, flash pulmonary oedema, medications, and baseline blood pressure, proteinuria and renal function. Revascularization exerted a 33% reduction in risk for death (hazard ratio 0.67 [95% confidence interval 0.52–0.87] *p* = 0.003) and a 32% reduction in risk for ESKD (hazard ratio 0.68 [95% confidence interval 0.53–0.88] *p* = 0.003); this is similar to the risk reduction noted in a recent observational study performed using administrative claims in Taiwan (adjusted odds ratio 0.64 [95% confidence interval 0.50–0.84] *p* < 0.01) [29]. It is however difficult to interpret the effect of revascularization on long-term outcomes in unselected patients with ARVD from observational or retrospective studies as these do not take into account hidden confounders or selection bias.

In addition to potential sources of bias already mentioned above, this study has other important limitations. Only patients with complete datasets were included in this analysis. The number of patients excluded from analysis due to missing data was small in comparison to the study population, and so we feel it unlikely that this would introduce potential bias in our study. Data was collected in a standardized manner from patient records, but this was performed by different individuals over three decades, thus introducing assignment bias. Variables such as body mass index, smoking status and drug dosage were not included due to missing or unreliable data. Cause of death data was also not available but for the purposes of our discussion, it was assumed that there was a predominance of cardiovascular deaths in this ARVD population, in keeping with evidence from the literature [34]. Our analyses are based on ‘all-cause’ death and no imputed outcome data was used in the analyses. Blood pressure was documented from office readings taken at time of diagnosis, which has limitations. The degree of stenosis was determined by a single observer and based on biplanar imaging studies without confirmation of haemodynamic significance of the stenosis. It is hoped that continued prospective data collection coupled with the application of novel non-invasive imaging techniques [35] and specific serum biomarkers [36], to determine the haemodynamic significance of a stenosis and the viability of renal parenchyma, can help overcome these limitations.

## Conclusion

The main determinants of adverse clinical outcomes in ARVD are prior cardiovascular disease and intra-renal parenchymal damage manifest by greater proteinuria and reduced renal function. Our results indicate that more effort is required to optimize medical management of ARVD using multi-targeted vascular protection therapy to help improve cardiovascular risk and decrease overall atherosclerotic burden while mitigating intra-renal parenchymal injury. Revascularization may have a beneficial effect on long-term outcomes in certain patients, however, more research is required to help characterize this patient sub-group further.

## Abbreviations

ARVD: Atherosclerotic renovascular disease
CHF: Congestive heart failure
CKD: Chronic kidney disease
CVE: Cardiovascular event
eGFR: Estimated glomerular filtration rate
ESKD: End-stage kidney disease
FPE: Flash pulmonary oedema
MVD: Macrovascular disease
RAB: Renin-angiotensin blockade
RAS: Renal artery stenosis
RRT: Renal replacement therapy
